# Supplementary material for: Role of Delocalization, Asymmetric Distribution of π-Electrons and Elongated Conjugation System for Enhancement of NLO Response of Open Form of Spiropyran-Based Thermochromes
Source: Molecules. 2023 Aug 28;28(17):6283. doi: 10.3390/molecules28176283 (PMC10488622; doi:10.3390/molecules28176283)
Supplement: Supplementary file 1 [file molecules-28-06283-s001.zip › molecules-2457680-supplementary.pdf]

Supplementary Information

Table S1. The ground state dipole moments (μ, in Debye), polarizability (α_o, in au) for close and open isomers of spiropyranes **1** & **2** in the presence of acetonitrile solvent.

Compounds	Isomer	μ	α_o
spiropyran **1**	close	8.94	373
	open	11.73	476
spiropyran **2**	close	8.36	553
	open	13.91	640

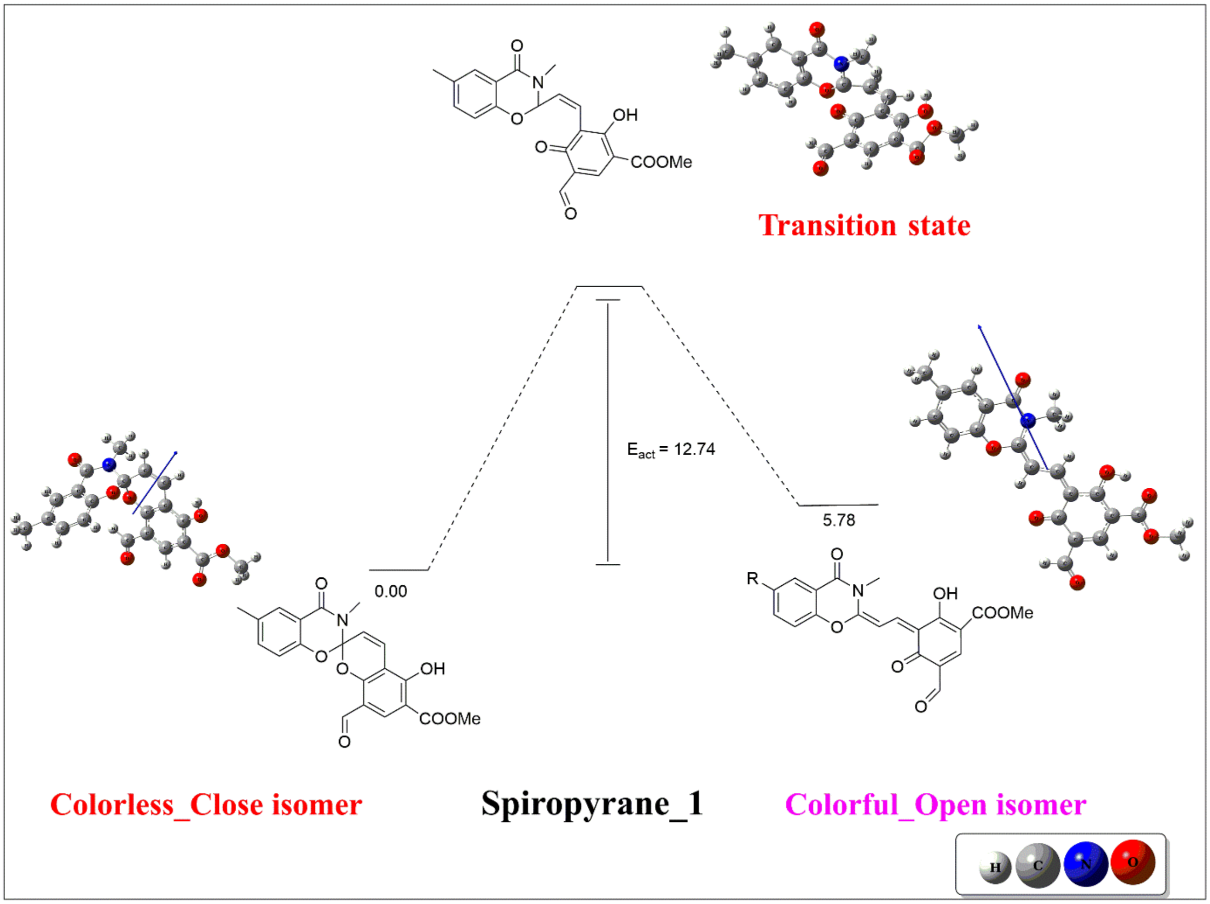

Figure S1. Optimized structures of close, transition state and open isomers of spiropyran **1**.

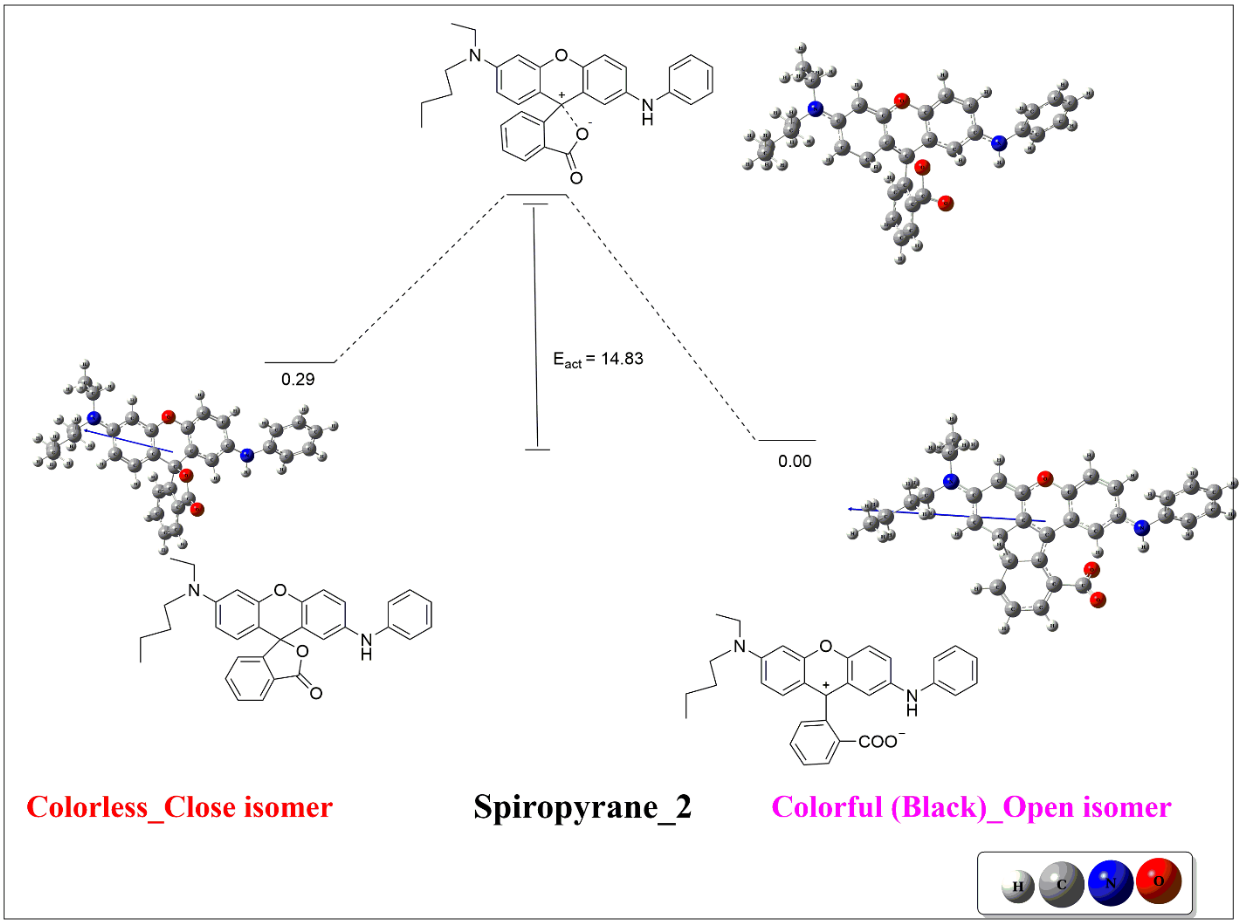

Figure S2. Optimized structures of close, transition state and open isomers of spiropyran **2**.

1 Cartesian coordinates of the optimized structures

1.1 Close isomer of spiropyran 1:

C	3.25639300	-2.93921100	-0.96341400
C	2.62262700	-2.17315900	0.00056700
C	1.28017500	-2.40643800	0.25841600
C	0.58316800	-3.39118700	-0.42207100
C	1.23923800	-4.14944400	-1.39131200
C	2.57870200	-3.93455500	-1.67960300
H	4.30567300	-2.75641700	-1.17374600
H	3.14591800	-1.39931300	0.54843400
C	-0.83769800	-3.64142600	-0.09374700
H	0.66992800	-4.91472800	-1.90733400

C	-0.72586500	-1.50748800	1.08857600
N	-1.39122500	-2.74922900	0.81773400
O	0.65839500	-1.66546300	1.22706700
O	-1.47049500	-4.56707900	-0.55575400
C	-2.83689200	-2.78693400	1.00962900
H	-3.32603200	-1.99746400	0.43220400
H	-3.18768000	-3.75473900	0.66044900
H	-3.08831000	-2.67194900	2.06437100
C	-0.72058500	0.65194100	0.03381700
C	-0.80911100	1.30015200	1.26853500
C	-1.18819200	0.49121800	2.41757200
C	-1.19732700	-0.83864200	2.34590000
H	-1.50736800	0.96609300	3.34079200
O	-0.97384200	-0.67218800	-0.05492800
C	-0.43175200	1.34722800	-1.14217000
C	-0.23070000	2.71879600	-1.04953900
C	-0.30503500	3.40867800	0.15384400
C	-0.57280000	2.68483700	1.32871400
H	0.01221800	3.26895600	-1.95172500
O	-0.57544200	3.33134900	2.50785300
C	-0.30578200	0.65428500	-2.44397600
O	-0.09892000	1.22991800	-3.48458400
H	-0.57806400	2.70286500	3.23161600
C	-0.04459800	4.87780700	0.08656700
O	-0.59682900	5.56451700	1.08642100
O	0.57290900	5.39297700	-0.81151200
C	-0.38062100	6.97591500	1.06235300
H	-0.86865500	7.35888900	1.95583000
H	0.68710800	7.19949200	1.08154100
H	-0.82053700	7.41593200	0.16582200
H	-0.40317900	-0.44484400	-2.41206900
C	3.29293300	-4.73422400	-2.73761000
H	4.21736600	-5.16748000	-2.34694700
H	2.66683700	-5.54855300	-3.10582800
H	3.55772500	-4.10315100	-3.59094200
H	-1.46826300	-1.47014900	3.18061600

1.2 Open isomer of spiropyran 1:

C	0.25473500	-4.19023300	5.07192900
C	0.09438200	-3.84985300	3.74216700
C	-0.15380600	-2.51971900	3.42866500
C	-0.24729300	-1.55692100	4.41571600
C	-0.08267800	-1.92236100	5.75399600
C	0.17404100	-3.23767300	6.10117400
H	0.45260600	-5.22639800	5.32714100
H	0.16359400	-4.58322800	2.94879000
C	-0.58931700	-0.18530000	4.03347700
H	-0.17053600	-1.14807900	6.50787700
C	-0.45012000	-0.92307700	1.69788600
O	-0.27841200	-2.19157900	2.11319400
C	0.37171700	-3.64913800	7.53550800
H	-0.27263700	-4.49286500	7.79498200
H	1.40652900	-3.95664600	7.71127800
H	0.14587400	-2.82733100	8.21652600
N	-0.75795400	0.02241600	2.64547900
O	-0.76616100	0.71798500	4.81366100
C	-1.31567200	1.32395400	2.27012100
H	-1.92468400	1.20508900	1.37614800
H	-1.92958200	1.66618100	3.09941600
H	-0.53187400	2.06295800	2.09831300
C	-0.33204300	-0.78200500	0.33354600
C	-0.16647300	0.41461000	-0.37926300
H	-0.27505400	-1.71221400	-0.21408200
C	-0.07211200	-0.57066100	-2.70079800
C	0.11280800	-0.23628300	-4.11343100
C	0.31519900	1.04996900	-4.50810800
C	0.36352600	2.14530400	-3.60549800
C	0.19730600	1.89836100	-2.24187300
C	-0.01972200	0.56344700	-1.74731700
H	0.44646500	1.24178700	-5.56823000
H	-0.08976100	1.34027100	0.17439500
O	0.23061300	2.87943300	-1.35149400
O	-0.25996700	-1.72752500	-2.32759900
C	0.07795900	-1.31263000	-5.12091400

O	0.21834000	-1.13022000	-6.31083400
H	0.38918900	3.72007100	-1.84882200
C	0.58340400	3.51256400	-4.05315500
O	0.63325100	4.47985800	-3.29664300
O	0.73107900	3.64396200	-5.36741400
C	0.96068600	4.96793800	-5.85481900
H	1.02731700	4.86964600	-6.93571900
H	0.13583800	5.62577900	-5.57856200
H	1.89137300	5.36620500	-5.44827000
H	-0.08809300	-2.32521900	-4.71657100

1.3 Close isomer of spiropyran 2:

C	-2.71148100	1.31358600	1.97791600
C	-1.65022500	1.53745500	1.13189500
C	-0.82908000	0.50602800	0.66848300
C	-1.13166400	-0.77522800	1.10872500
C	-2.18929700	-1.03345100	1.97183300
C	-3.01294200	0.00409700	2.42633600
C	0.70919900	-1.69975500	-0.02110700
C	1.10944800	-0.47538700	-0.53278900
C	2.26531400	-0.42251400	-1.31565000
H	2.56825100	0.52939100	-1.74090900
C	3.02722100	-1.55266900	-1.56761100
C	2.61200500	-2.77650900	-1.02165700
C	1.45987500	-2.84887500	-0.26918700
H	-3.32196300	2.15437100	2.27569400
H	-2.32815400	-2.05924800	2.28206800
H	3.20968500	-3.66560800	-1.18255400
H	1.12963000	-3.78838700	0.15696800

O	-0.40483300	-1.86896700	0.74614800
C	1.13499500	1.96331500	0.08523300
C	1.03944500	2.92587700	-0.89677900
C	1.71730700	4.13486500	-0.81068000
C	2.50592200	4.34769800	0.31100200
C	2.60246400	3.37229700	1.30992500
C	1.91740900	2.16707900	1.21076800
C	0.28805700	0.76946200	-0.29872800
C	0.13451400	2.42138800	-1.95721200
H	1.62360600	4.87469800	-1.59708100
H	3.05315400	5.27691400	0.42029800
H	3.22249900	3.56337200	2.17879500
O	-0.28119700	1.18657000	-1.58776400
O	-0.20871100	2.95084000	-2.97428900
N	4.22307000	-1.45128100	-2.29190600
H	4.64900300	-0.53942500	-2.29518100
C	4.63653900	-2.30247700	-3.32273800
C	5.95208800	-2.19253200	-3.78816200
C	6.40412800	-2.99752300	-4.82163600
C	5.56353900	-3.94211700	-5.40184600
C	4.25832700	-4.05412100	-4.94028300
C	3.78813600	-3.24013900	-3.91724700
H	6.62030200	-1.47273400	-3.32578900
H	7.42605400	-2.89154200	-5.16894800
H	5.92038300	-4.57779700	-6.20330500

H	3.58446000	-4.77443900	-5.39117300
H	2.75720800	-3.32104300	-3.59548100
H	1.99043800	1.41379700	1.98698600
H	-1.45223900	2.55196200	0.80089800
N	-4.06694100	-0.23721000	3.27508400
C	-4.85638100	0.84161000	3.83999300
C	-6.01950200	1.28406500	2.95093300
C	-3.73583700	-2.17084000	4.81383900
C	-6.82694300	2.42400000	3.56541000
C	-8.01154400	2.84285900	2.69943400
C	-4.47774400	-1.58896300	3.61186300
H	-5.55169200	-1.56362300	3.81847500
H	-4.36015300	-2.23368200	2.73649700
H	-4.09103400	-3.18170500	5.03216300
H	-2.66033500	-2.21555000	4.62877600
H	-3.89806900	-1.55351100	5.70151600
H	-5.24455200	0.49831800	4.80510900
H	-4.20814300	1.69203100	4.07111300
H	-5.63129100	1.58542500	1.97171000
H	-6.67375100	0.42276300	2.76947400
H	-7.18686500	2.12027300	4.55603500
H	-6.16983400	3.28630600	3.73029100
H	-8.69443900	2.00315000	2.53896100
H	-8.58015500	3.65197100	3.16442400
H	-7.67672300	3.18994300	1.71750400

1.4 Open isomer of spiropyran 2:

C	-0.56513100	0.20171200	3.50800500
C	-0.62981600	0.81244400	2.18198500
C	-0.19096400	-0.00637100	1.06049300
C	0.09704900	-1.36235100	1.21016100
C	0.13969200	-1.88960400	2.47669600
C	-0.20131500	-1.12883600	3.66075200
C	0.45966400	-1.48145200	-1.10788200
C	0.28458000	-0.10231400	-1.30423700
C	0.16626500	0.37238000	-2.60683100
H	-0.07863400	1.40980200	-2.78696300
C	0.31210100	-0.47070300	-3.71235400
C	0.54993300	-1.83523800	-3.47884200
C	0.60341800	-2.33181200	-2.18940200
H	-0.95224100	0.77016200	4.34138000
H	0.50086500	-2.90540500	2.57225800
H	0.68707600	-2.51090100	-4.31281200
H	0.76381200	-3.38798700	-2.01128000
O	0.51120700	-2.07446300	0.15124900
C	0.26743000	2.05048700	0.27638000
C	0.39935800	3.25706300	-0.48988100
C	-0.02065100	4.42785000	0.06557900
C	-0.50272400	4.52114200	1.42086900
C	-0.42329300	3.46887500	2.25238900
C	0.12380600	2.17904300	1.77454000
C	0.14341600	0.71423900	-0.09544400
C	0.97294700	3.32192400	-1.87937100
H	0.01030300	5.32571900	-0.54221200
H	-0.86779800	5.47734600	1.77511600
H	-0.68874100	3.56225600	3.29984600
O	0.46482100	3.93271900	-2.77214900
N	0.22817100	0.07299100	-4.97551900
H	0.39640000	1.06403700	-5.04309200
C	0.24599800	-0.64418700	-6.19455000
C	1.14601200	-0.27939600	-7.19325700
C	1.13505700	-0.94050400	-8.41501600
C	0.24472600	-1.98309500	-8.63998700

C	-0.64965100	-2.35007300	-7.64025900
C	-0.66211400	-1.67679100	-6.42645500
H	1.85567000	0.52052600	-7.00983800
H	1.83355600	-0.64451400	-9.18908500
H	0.24189500	-2.50152000	-9.59127400
H	-1.35979700	-3.15044100	-7.81363500
H	-1.38752300	-1.93649900	-5.66347200
H	1.11169200	2.03588400	2.23102400
H	-1.68626200	1.06025000	1.99250800
N	-0.15722300	-1.74689300	4.87160100
C	-0.32842600	-0.98572100	6.10235600
C	-1.78478100	-0.83510100	6.54170000
C	1.48802800	-3.60425800	5.15175700
C	-1.91666000	-0.03098000	7.83300000
C	-3.36637300	0.13413300	8.27856100
C	0.02566000	-3.18912000	5.02630400
H	-0.52232500	-3.48325300	5.92447500
H	-0.46720800	-3.71428400	4.20453000
H	1.56519000	-4.68221500	5.30937400
H	2.05982600	-3.34917100	4.25572300
H	1.95981200	-3.10434800	6.00101100
H	0.23875100	-1.49851200	6.88445300
H	0.14643200	-0.00575900	5.98728900
H	-2.36838800	-0.35889900	5.74513500
H	-2.22045400	-1.83116400	6.68063500
H	-1.34261300	-0.52498500	8.62523300
H	-1.45891200	0.95630900	7.69619900
H	-3.84148400	-0.83781300	8.43830000
H	-3.43128800	0.69377100	9.21378200
H	-3.95192700	0.67107300	7.52644900
O	2.13981700	2.68294200	-2.06027100
